# The Pentose Phosphate Pathway and Its Involvement in Cisplatin Resistance

**DOI:** 10.3390/ijms21030937

**Published:** 2020-01-31

**Authors:** Isabella Giacomini, Eugenio Ragazzi, Gianfranco Pasut, Monica Montopoli

**Affiliations:** 1 Department of Pharmaceutical and Pharmacological Sciences, University of Padua, Largo Egidio Meneghetti 2, 35131 Padova, Italy; isabella.giacomini@studenti.unipd.it (I.G.); eugenio.ragazzi@unipd.it (E.R.); 2 Department of Pharmaceutical and Pharmacological Sciences, University of Padua, Via Marzolo 5, 35131 Padova, Italy; gianfranco.pasut@unipd.it; 3 Veneto Institute of Molecular Medicine, Via Giuseppe Orus 2, 35129 Padova, Italy

**Keywords:** cisplatin, pentose phosphate pathway, cancer, resistance, drug delivery systems

## Abstract

Cisplatin is the first-line treatment for different types of solid tumors, such as ovarian, testicular, bladder, cervical, head and neck, lung, and esophageal cancers. The main problem related to its clinical use is the onset of drug resistance. In the last decades, among the studied molecular mechanisms of cisplatin resistance, metabolic reprogramming has emerged as a possible one. This review focuses on the pentose phosphate pathway (PPP) playing a pivotal role in maintaining the high cell proliferation rate and representing an advantage for cancer cells. In particular, the oxidative branch of PPP plays a role in oxidative stress and seems to be involved in cisplatin resistance. In light of these considerations, it has been demonstrated that overexpression and higher enzymatic activity of different enzymes of both oxidative and non-oxidative branches (such as glucose-6-phosphate dehydrogenase, 6-phosphogluconate dehydrogenase, and transketolase) increase cisplatin resistance, and their silencing or combined treatment with cisplatin could restore cisplatin sensitivity. Moreover, drug delivery systems loaded with both PPP inhibitors and cisplatin give the possibility of reaching cancer cells selectively. In conclusion, targeting PPP is becoming a strategy to overcome cisplatin resistance; however, further studies are required to better understand the mechanisms.

## 1. Introduction

*Cis*-diamminedichloroplatinum (II), also known as *cis*-platinum or cisplatin (CDDP), synthesized in 1844 by Michele Peyrone, is still considered as first-line treatment for many human solid tumors, such as ovarian, testicular, or head and neck cancers [1,2,3,4,5,6].

Despite its use as first-line treatment, one of the main factors that hamper the effectiveness of the therapy is the onset of drug resistance, which leads to tumor relapse and clinical failure [3,6]. Moreover, another important issue of cisplatin clinical use is related to its severe toxicity, such as ototoxicity, gastrotoxicity, myelosuppression, nephrotoxicity, and allergic reactions [7].

Various molecular mechanisms have been described as potentially responsible for the development of cisplatin resistance. Reduced intracellular drug accumulation represents the 70–90% of resistance and it could be caused by an inhibition of drug uptake or increased drug efflux [8,9]. The formation of adducts with glutathione (GSH), metallothioneins, and other “scavengers” with nucleophilic properties is another well-known mechanism of resistance; in fact, in different types of solid tumors, the sequestration by these nucleophilic species has been demonstrated to inactivate the drug leading to increased cellular survival [6]. Considering that cisplatin targets DNA, active DNA-damage repair systems such as nucleotide excision repair (NER) system and the mismatch repair (MMR) machinery, are involved in reactivation of cell cycle and tumor growth maintaining cell viability [10]. Many studies showed that the consequence of such mechanisms of resistance is attenuated apoptosis [6,9,11].

During the last decades, several studies demonstrated that the metabolic reprogramming of tumor cells, which is already known as a hallmark of cancer, has been playing an important role in the onset of chemotherapy resistance [12,13]. Cancer cells reprogram their metabolism rewiring their biological pathways in order to maintain a higher proliferation rate, to increase invasion and migration, to avoid apoptosis and growth suppressors, and to induce angiogenesis [14]. In other words, metabolic reprogramming involves a series of metabolic alterations, which can interest all the principal pathways from glucose metabolism to glutamine and lipids, and mitochondrial alterations [15].

Considering the above-mentioned mechanisms, a great deal of interest has been paid to the study of metabolic alterations occurring in resistant cells with the final aim to identify specific targets exploitable to overcome drug resistance. The main difficulty is to find a safe therapeutic window between high proliferative cancer cells and normal ones [16,17,18].

In this review, we will focus our attention on metabolic reprogramming, particularly on the pentose phosphate pathway (PPP) and its involvement in the onset of cisplatin resistance.

## 2. Glucose Metabolism and the Pentose Phosphate Pathway in Tumor Cells

During the last decades, glucose metabolism has gained a lot of interest in its role in cancer and many studies have demonstrated the importance of glucose for cellular survival and the development of drug resistance [19].

The “Warburg effect”, one of the most recognized alterations in cancer metabolism, affects glucose metabolism and was firstly described in the 1920s by Otto Warburg. It consists of a shift of cellular metabolism from oxidative phosphorylation toward aerobic glycolysis, even in the presence of oxygen, with increased lactate and ATP production and increased glucose uptake [16,20]. In fact, cancer cells rely on glycolysis as the major source of ATP, and then of energy [12]. Moreover, the Warburg effect may represent an advantage for cancer cells, thanks to the microenvironment acidification caused by increased lactate production [20].

Cancer cells use glucose metabolism in order to supply the higher required proliferation rate, and, in particular, PPP is a metabolic pathway used both to synthesize nucleic acids and to maintain the cellular redox state [21,22].

**Figure 1:**
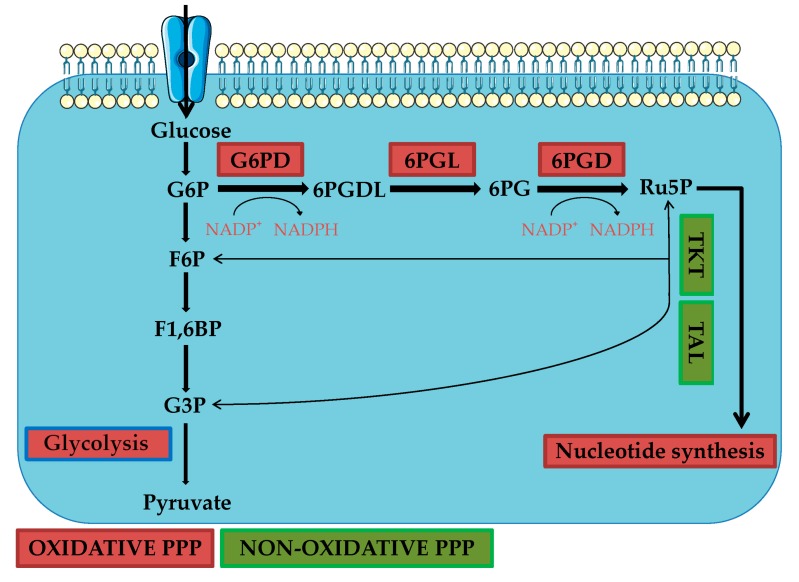
The pentose phosphate pathway (PPP) is a metabolic pathway involved in the onset of cisplatin resistance. The oxidative branch (red frames) comprises three reactions: the first one involves the enzyme glucose 6-phosphate dehydrogenase (G6PD); the second reaction is catalyzed by phosphogluconolactonase (6PGL); the third reaction involves the enzyme 6-phosphogluconate dehydrogenase (6PGD). The non-oxidative branch (green frames) comprises other reactions and the enzymes involved are transketolase (TKT) and transaldolase (TAL).

The PPP (Figure 1), also known as hexose monophosphate shunt or phosphogluconate pathway, is a branch of glycolysis and it represents an important step in glucose metabolism, using glucose-6-phosphate (G6P) as the primary substrate [23]. The PPP is used by cells to synthesize ribonucleotides and nicotinamide adenine dinucleotide phosphate (NADPH), the latter essential for reductive biosynthesis, such as that for lipid production [19,24]. It is also known that NADPH plays a central role in the cellular redox state. In particular, different studies demonstrated the crucial role of PPP in cancer cells, in the onset of drug resistance, and also in cisplatin resistance [24,25,26]. In cancer cells a hyperactive PPP has been found, since they use more pentose phosphates to enhance nucleic acids synthesis, necessary to supply the higher proliferation rate. Additionally, activation of PPP counteracts oxidative stress, thus allowing cancer cells to develop drug resistance [26]. The proposed mechanism involved in the onset of drug resistance is related to Reactive Oxygen Species (ROS) production. In fact, it is known that cellular redox state is different in cancer cells compared to normal ones underlying the correlation between altered homeostasis and tumor development and progression. Several studies demonstrated that cancer cells have to monitor ROS production in order to maintain their high proliferation rate. In light of the fact that tumor cells present higher ROS levels relative to normal cells, two hypotheses have been proposed. The first one considers high ROS levels useful to enhance pro-oncogenic mutations and pathways. The second one refers to the fact that high ROS levels are correlated with higher oxidative stress. Consequently, cancer cells present overactivated PPP to produce NADPH used to counteract ROS damage, yielding cancer resistance [21]. Moreover, cancer cells take advantage of PPP using NADPH to synthesize more fatty acids and to survive under stress conditions [21].

The PPP is divided into two biochemical branches: the oxidative one and the non-oxidative branch and it involves a series of different reactions occurring in the cytosol.

Starting from the oxidative branch, the first reaction consists of the irreversible conversion of G6P into 6-phoshogluconolactone (6-PGDL), catalyzed by one of the most important enzymes of this pathway, the glucose-6-phosphate dehydrogenase (G6PD). From this reaction, the first molecule of NADPH is produced. G6PD is overexpressed and has higher activity in different types of tumors, like ovarian, lung, renal, and oral cancer [25,26,27,28]. Moreover, its knockdown or use of inhibitors is able to decrease tumor growth [29,30]. The second reaction, catalyzed by phosphogluconolactonase (6PGL), hydrolyzes 6-PGDL into 6-phosphogluconate (6-PG). The third reaction consists of an oxidative decarboxylation of 6-PG into ribulose-5-phosphate (Ru5P), catalyzed by 6-phosphogluconate dehydrogenase (6PGD), and generates a second molecule of NADPH. 6PGD is overexpressed in different tumors, such as ovarian and lung cancer [31,32]. The most important role of the oxidative PPP branch is to protect cells from oxidative stress and, in order to do it, NADPH is used as a scavenger against ROS [27,33]. Conversely, cancer cells also consume NADPH during fatty acids synthesis (FAS) [27].

The non-oxidative branch includes various reversible reactions, consisting of the conversion of different glycolytic intermediates such as fructose-6-phosphate (F6P) and glyceraldehyde-3-phosphate (G3P), into pentose phosphate and vice versa [21,22]. The main enzymes involved in non-oxidative PPP are transketolase (TKT) and transaldolase (TALDO). Transketolase is responsible for the conversion of ribose-5-phosphate (R5P) and xylulose-5-phosphate (Xu5P) into G3P and sedoheptulose-7-phosphate (S7P), respectively. Transaldolase is responsible for transferring a C3 unit from S7P to G3P forming erythrose-4-phosphate (E4P) and fructose-6-phosphate (F6P) [21]. The increased expression of TKT is related to tumors [34].

## 3. Targeting PPP Could Be a Strategy to Overcome Cisplatin Resistance

The pentose phosphate pathway seems to be involved in the response to the onset of cisplatin resistance and many studies have been conducted to verify if targeting specific enzymes or specific products of the oxidative and non-oxidative branches of PPP could be a strategy to overcome cisplatin resistance.

Catanzaro et al. [25] demonstrated that G6PD is overexpressed in cisplatin-resistant cancer cells (C13), compared to their sensitive counterpart (2008). A combined treatment of cisplatin and 6-aminonicotinamide (6-AN), a competitive G6PD inhibitor, was tested demonstrating a sensitization of cisplatin-resistant cells. Therefore, it was suggested that targeting G6PD could become a strategy to overcome cisplatin resistance [25]. The higher expression and activity of G6PD is also verified on another ovarian cancer cell line, also cisplatin resistant, SKOV3/DDP. The treatment of cisplatin-resistant cells with an uncompetitive inhibitor of G6PD, the dehydroepiandrosterone (DHEA), or the competitive inhibitor 6-AN in combination with cisplatin, reduced cell viability more than cisplatin alone [35]. Lucarelli et al. [36] underlined an increased expression and a higher enzymatic activity of G6PD in renal cisplatin-resistant cancer cells, ccRCC. ccRCC pretreated with 6-AN and then treated with cisplatin showed a higher death rate in comparison to mono treatment with cisplatin, thus further supporting that the inhibition of G6PD could be involved in the sensitization of cells to cisplatin [36]. Targeting G6PD to overcome cisplatin resistance could also be a strategy in non-small-cell lung cancer (NSCLC). In fact, this enzyme is overexpressed and shows higher activity in A459/DDP. In their work, Hong et al. [26] tested a combination of cisplatin and 6-AN or used si*G6PD*, a siRNA, to silence the enzyme. Results showed decreased expression and activity of G6PD, thus sensitizing cisplatin-resistant cells to the drug [26]. Recently, Zhang and coworkers [37] demonstrated that the enzyme G6PD is overexpressed by the transforming growth factor beta 1 (TGF β1) through the activation of the forkhead box protein M1-high mobility group AT-hook 1-G6PD (FOXM1-HMGA1-G6PD) transcriptional regulatory pathway. Moreover, they identified the FOX1-HMGA1-TGF β1 pathway as responsible for cisplatin resistance and interfering with it represents a strategy to sensitize NSCLC cells [37]. Mele et al. [38] tested the combined treatment of cisplatin and polydatin, natural inhibitor of G6PD, in an orthotopic xenograft model of oral cancer. Data showed a synergic effect of the molecules, in inducing cytotoxicity on cancer cells, in reducing tumor growth, and in inhibiting lymph node metastasis [38].

The enzymatic activity and expression of 6PGD are upregulated in cisplatin-resistant cells, both ovarian (C13) and lung (A459DDP) cancers. Zhang et al. [31] demonstrated that treating cells with 1-Hydroxy-8-methoxy-anthraquinon (S3), an inhibitor of cancer cell proliferation and cellular growth caused a decrease in the enzymatic activity of 6PGD, not correlated with a decrease in protein expression. Moreover, the combined treatment between S3 and cisplatin produced a synergistic effect, leading to a resensitization of cisplatin-resistant cells, both C13 and A459DDP [31]. In their work, Zheng et al. [32] analyzed the correlation between the higher expression and activity of the enzyme and the reduced survival of patients. Moving deeply into molecular mechanisms of resistance, they tested, in both C13 and A459DDP cells, the combined treatment of physcion (a natural dihydroxyanthraquinone) and cisplatin which resulted in the sensitization of cisplatin-resistant cells to the drug. Moreover, they demonstrated that miR-206 or miR-613 is downregulated in cisplatin-resistant cancer cells and their transfection into cells caused a decreased expression and lower enzymatic activity of 6PGD. Thus, the treatment with miR-206 or miR-613 sensitizes resistant clones (C13 and A459DDP) to CDDP, while conferring resistance to the sensitive ones (OV2008 and A459) [32].

Considering the non-oxidative branch, there are fewer studies in the literature. Yang et al. [39] identified that inhibition or genetic silencing of TKT improves cisplatin sensitivity in cervical cancer HeLa cells. In their study, they demonstrated that TKT is a direct target of miR-497 and that treatment with miR-497 sensitizes cervical cells to cisplatin by decreasing TKT expression [39]. Dong and Wang [40] demonstrated that human nasopharyngeal carcinoma (NPC) cell lines CNE1 and HONE1, present increased levels of transketolase-like protein 1 (TKTL1). Moreover, the knock-down of TKTL1 decreases NADPH and R5P levels and the combined treatment of TKTL1 knock-down and CDDP decreases cell viability in both cell lines [40].

Micro-RNAs are involved in cell growth and other processes, like migration or invasion. In particular, miR-222 upregulation and its association with modulation of response to cancer therapy have been reported in various human cancers [41,42]. Zeng and co-workers [43] demonstrated that miR-222 overexpression enhances the survival rate of T24 and 5637 bladder cancer cells treated with cisplatin. Moreover, they indicate that miR-222 activates the Akt/mTOR axis and directly targets the protein phosphatase 2A subunit B (PPP2R2A), reducing cisplatin-induced autophagy [43]. Recent studies demonstrated a correlation between the overexpression or the downregulation of mi-RNAs and the onset of chemoresistance [41,42]. For example, as already mentioned above, the miR-613 and miR-206 downregulation are related to cisplatin resistance. Moreover, it has been characterized that these mi-RNAs play an important role in cisplatin resistance because their regulation is correlated with the upregulation of 6PGD, underlying the strict involvement of mi-RNAs in chemoresistance and their consequent involvement in regulating metabolism. Anyway, further studies will be necessary to confirm the involvement of mi-RNAs in the onset of cisplatin resistance through the PPP regulation [32].

Oxidative stress is implicated in the onset of cisplatin resistance. In fact, there is an increased ROS production in cancer cells because of mitochondria dysfunction or altered metabolism and, consequently, there is enhanced production of antioxidant molecules [44]. The combined treatment of 2-deoxy-d-glucose (2-DG) and cisplatin increases the oxidative stress and then the cytotoxicity in FaDu human head and neck cancer cells [45].

**Figure 2:**
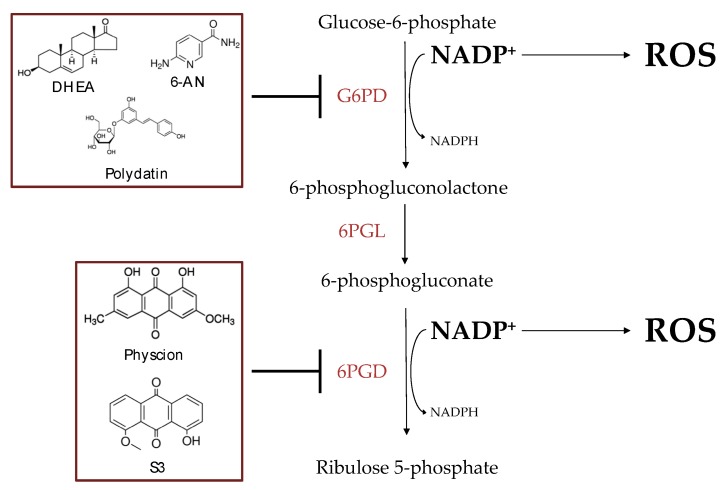
Graphical view of the metabolic and molecular effects of the combined treatment with PPP inhibitors and cisplatin (CDDP). The symbol ――| indicates inhibition of the enzymes G6PD and 6PGD. The inhibition of the enzyme leads to increased ROS accumulation and resensitization of CDDP-resistant cells. G6PD: glucose 6-phosphate dehydrogenase; 6PGL: phosphogluconolactonase; 6PGD: 6-phosphogluconate dehydrogenase.

One of the proposed molecular mechanisms involved in the resensitization of cisplatin-resistant cells treated with PPP inhibitors seems to be ROS generation (Figure 2). In fact, G6PD is the enzyme that catalyzes the production of NADPH, used by cells to counteract ROS production. The ability of cisplatin to kill cells is not only due to DNA damage, but also to the increase in oxidative stress caused by enhanced ROS levels. Considering that the overexpression of G6PD increases the NADPH production and it is related with cisplatin resistance, the inhibition of this enzyme leads to a chemotherapy resensitization decreasing the production of NADPH and losing the ability to counteract ROS accumulation [21,26]. Moreover, 6PGD is the third enzyme of PPP and it produces the second molecule of NADPH. The inhibition of 6PGD is also involved in the redox homeostasis [29].

Considering the above-reported results, many studies demonstrated that targeting different enzymes of both oxidative and non-oxidative branches could become a strategy to overcome cisplatin resistance (Table 1). However, further investigations will be necessary in order to identify new pharmacological targets useful to counteract cisplatin resistance.

## 4. Drug Delivery Systems Targeting PPP to Overcome Cisplatin Resistance

As reported above the inhibition of the PPP pathway can sensitize cancer cells to anticancer drugs, thus helping to overcome drug resistance. Drug delivery systems can help to deliver a higher amount of active cisplatin, or other anticancer drugs, to the tumor and, at the same time, can reduce the side effects of chemotherapy by a selective targeting. Drug delivery systems allow accumulation of drugs into tumors thanks to two mechanisms: (I) the exploitation of enhanced permeability and retention effect of cancers which is the consequence of the abnormal angiogenic stimulus [46], and (II) the ligand-mediated targeting, achieved by linking to drug delivery systems a targeting agent that is able to bind a marker overexpressed on the surface of cancer cells [47].

In this view, a liposomal formulation of cisplatin was studied for prolonged pharmacokinetics of the drug in combination with 6-AN, an inhibitor of the enzyme G6PD (the rate-limiting step of the PPP), to restore the sensitivity of resistant cisplatin ovarian cancer cells [48]. In this case, the cisplatin loaded liposomes were vesicles with polyethylene glycol chains anchored on the surface for conferring a stealth property to the liposomes, and thus further prolonging their pharmacokinetics. The combination of 6-AN and liposomal cisplatin showed promising cytotoxic effects in resistant cells [48]. On the other hand, as for anticancer drugs, the delivery of PPP inhibitors should be selectively, or at least preferentially, directed at the site of action thus allowing the reduction of potential side effects. Consequently, future development in this direction would be a delivery system able to incorporate both the PPP inhibitor and the anticancer drug. The simultaneous loading of the inhibitor and the drug on the same delivery system will ensure a matched body fate for the two molecules, owing to the identical pharmacokinetics and biodistribution, thus enhancing significantly the therapeutic effectiveness. Moreover, such an approach would allow the selection of the proper drug/inhibitor ratio, maximizing the combination outcome. Although feasible, such a combination might not be suitable for certain drugs or drug delivery systems. For example, in the above-mentioned approach of liposomal cisplatin, the concomitant loading of 6-AN into the same liposomes induced cisplatin degradation [48]. It is therefore mandatory to investigate the compatibility of the drug and the inhibitor and possibly when the two cannot come into contact, it would be necessary to direct the development towards two separate, but similar, drug delivery systems: one for the inhibitor and one for the drug. Alternatively, a drug delivery system can be investigated in which the two molecules are physically entrapped and not present in solution, like polymeric nanoparticles. The instability issue is related to such specific combination of cisplatin and 6-AN, because in other studies cisplatin has been loaded inside liposome (e.g., with valproate or curcumin) without problems of instability [49].

The selection of the drug delivery system is also dictated by the intrinsic cytotoxic activity of the drug. A very potent cytotoxic agent, with subnanomolar potency, can be delivered through the antibody-drug conjugate format, in which the antibody plays the role of a carrier and a targeting agent. Differently, less active molecules require a drug delivery system that can carry a higher amount of drug molecules, like a liposome or a nanoparticle, if necessary, functionalized on the surface with a monoclonal antibody for selective targeting. For cisplatin, the second option is more suitable because the entrapment inside a liposome can protect the drug from degradation.

Additionally, the Warburg effect, the alteration of glucose metabolism in cancer cells, can be an interesting target for promoting the overcome of drug resistance in cancer cells. In has been demonstrated that nitric oxide can increase the antitumor activity of several chemotherapeutics, while it provides protection against apoptosis induced by oxidative stress in non-neoplastic cells [50]. The potential of the combination of the activity of nitric oxide with epirubicin was tested by linking a nitric oxide donor and the drug to a polymeric carrier, poly(ethylene glycol), obtaining a polymer conjugate that ensured the same biodistribution and cell internalization for the two active molecules. Among the several conjugates studied the one that carried the higher nitric oxide/epirubicin ratio, about 16 nitric oxide units per epirubicin molecule, displayed greater activity in Caco-2 cells while it decreased toxicity against endothelium cells and cardiomyocytes with respect to free epirubicin [51]. The antitumoral activity was investigated *in vivo* in Caco-2 and SKOV-2 tumor-bearing mice by the measurement of tumor diameters and weights. In comparison with free epirubicin and pegylated epirubicin, the conjugate bearing simultaneously epirubicin and the nitric oxide donor showed more potent antineoplastic effects, as demonstrated by the 95% reduction of tumor volume. Moreover, while administration of epirubicin and pegylated epirubicin resulted in the development of severe anthracycline cardiomyopathy, the mice treated with the conjugate with both drugs did not show any clinical and biochemical signs of cardiotoxicity [52].

Consequently, the possibility to selectively deliver into the same cancer cell an anticancer drug and a PPP inhibitor or drug affecting the glucose metabolism seems a strategy with great potential for overcoming the issue of drug resistance.

## 5. Conclusions

In this review, we focused on a specific pathway of cancer metabolism, the pentose phosphate pathway (PPP), and its involvement in the onset of cisplatin resistance. As already mentioned above, PPP comprises the oxidative and non-oxidative branches, which consist of a series of different reactions catalyzed by different enzymes. Among them, studies identified G6PD, 6PGD, and TKT as possible targets to overcome cisplatin resistance. Besides molecular strategies, such as mi-RNAs or genetic silencing, different enzymatic inhibitors have been tested, alone or in combined treatment with CDDP, and the results are promising. Moreover, drug delivery systems have been developed in order to target cancer cells selectively. In particular, PPP inhibitors and CDDP have been loaded in liposomal formulations in order to directly affect tumor cells leading to the sensitization of cisplatin-resistant cells.

In light of these observations, further studies are necessary to better understand the molecular mechanisms linking resistance to the reprogramming of the PPP pathway. Improving the knowledge of these interconnections may help in identifying new pharmacological targets exploiting PPP, besides the possibility to take advantage of drug delivery systems to reach cancer cells selectively, reducing CDDP toxicity.

## Figures and Tables

**Table 1:** Brief summary of the principal enzymes involved in cisplatin resistance and possible pharmacological inhibitors/molecular strategies able to sensitize resistant cells.

Enzymes	Therapeutic/Molecular Strategies	Experimental Model	References
G6PD overexpression	6-aminonicotinamide (6-AN) (competitive inhibitor)	Ovarian cancer cells: C13, IGROV Pt and SKOV3DDP	[25,35,48]
		Renal cancer cells: ccRCC	[36]
		Non-small-cell lung cancer: A459/DDP	[26]
	Dehydroepiandrosterone (DHEA) (uncompetitive inhibitor)	Ovarian cancer cells: SKOV3DDP	[35]
	Polydatin (natural inhibitor)	Orthotopic xenografts model of oral cancer	[38]
	Genetic silencing	Non-small-cell lung cancer: A459/DDP	[26]
6PGD overexpression	1-hydroxy-8-methoxy-anthraquinon (inhibitor of cancer cell proliferation and growth)	Non-small-cell lung cancer: A459/DDP Ovarian cancer cells: C13	[31] [31]
	Physcion (natural dihydroxyanthraquinone)	Non-small-cell lung cancer: A459/DDP Ovarian cancer cells: C13	[32] [32]
	Transfection with miR-206 or miR-613	Non-small-cell lung cancer: A459/DDP Ovarian cancer cells: C13	[32] [32]
TKT overexpression	Genetic silencing or miR-497 treatment	Cervical cancer cells: HeLa	[39]
